# Switch from intravenous or intramuscular to subcutaneous hepatitis B immunoglobulin: effect on quality of life after liver transplantation

**DOI:** 10.1186/s12955-020-01349-5

**Published:** 2020-04-10

**Authors:** Riccardo Volpes, Patrizia Burra, Giacomo Germani, Matteo Angelo Manini, Lucio Caccamo, Paolo Strignano, Giorgia Rizza, Mariarosa Tamè, Antonio Daniele Pinna, Fulvio Calise, Carla Migliaccio, Paola Carrai, Paolo De Simone, Maria Filippa Valentini, Luigi Giovanni Lupo, Gabriella Cordone, Francesco Paolo Picciotto, Antonio Nicolucci

**Affiliations:** 1 Mediterranean Institute for Transplantation and High Specialization Therapies, Palermo, Italy; 2 Multivisceral Transplant Unit (Gastroenterology), Department of Surgery, Oncology and Gastroenterology,Surgical and Gastroenterological Sciences, Padua University Hospital, Padua, Italy; 3 1st Division of Gastroenterology and Hepatology, Ca’ Granda-Maggiore Polyclinic Hospital, Scientific Institute for Research, Hospitalization, and Health Care, Milan, Italy; 4 General Surgery and Liver Transplant Center, S. Giovanni Battista Hospital, Turin, Italy; 5 Liver and Multiorgan Transplantation, Department of General Surgery, Sant’Orsola-Malpighi Polyclinic, Bologna, Italy; 6 Hepatobiliary Surgery and Transplant Unit, Cardarelli Hospital, Naples, Italy; 7 Department of General Surgery and Liver Transplantation, University of Pisa Medical School Hospital, Pisa, Italy; 8 General Surgery and Liver Transplantation Unit, Department of Emergency and Organ Transplantation, University of Bari, Bari, Italy; 9 Hepatology Unit, AORN A. Cardarelli, Naples, Italy; 10 Center for Outcomes Research and Clinical Epidemiology, Via Tiziano Vecellio, 2, 65124 Pescara, Italy

**Keywords:** Questionnaire, Satisfaction, Acceptability of treatment, Side effects, Patient autonomy

## Abstract

**Background:**

Hepatitis B immunoglobulin (HBIG) therapy is available in intravenous (IV) or intra-muscular (IM) formulations. Recently, a subcutaneous (SC) formulation was introduced. This study evaluated changes in quality of life when liver transplant (LT) recipients were switched from IV or IM HBIG to the SC formulation.

**Methods:**

This multicentre, observational study involved adults who had undergone LT at least 1 year prior to study entry. Quality of life was evaluated using the ITaLi-Q questionnaire, assessing the impact of HBIG therapy on daily activities and patient satisfaction, and the SF-36 Health Survey. Patients completed the questionnaires prior to switching from IV or IM HBIG to SC HBIG and 6 months later.

**Results:**

Eighty-six patients were enrolled; before the switch, 68.6% were receiving IM HBIG and 31.4% IV HBIG. After 6 months, significant improvements in 7 of the 8 ITaLi-Q domains were found, particularly side effects, need for support to adhere to the therapy and satisfaction with the HBIG therapy. Significant improvements in several SF-36 domains were documented, including physical functioning, physical and emotional role limitations, pain, social functioning, physical and mental summary scores.

**Conclusions:**

The SC route of administration reduces side effects and their interference with daily life, ameliorates negative feelings, and increases patient autonomy.

## Background

Hepatitis B virus (HBV) infection is a major cause of cirrhosis, hepatocellular carcinoma and end stage liver disease. When indicated, in these conditions liver transplantation (LT) is the only definitive treatment, and HBV-related disease accounts for almost 13% of all indications for LT in Europe [1]. In recent years, survival rates following LT have increased markedly following improvement in surgical techniques, advances in immunosuppressive therapy and management of post-operative complications [2]. In addition, HBV-positive recipients have benefited from use of antiviral drugs and immunoglobulin therapies to prevent HBV reinfection of the graft [2].

The recommendations of most of the international assotciations for the Study of the Liver endorsed a prophylaxis with hepatitis B immunoglobulin (HBIG) to prevent post-transplant HBV reinfection [3–5]. It is thus essential that its use be as convenient and acceptable to patients as possible to avoid non-adherence to the regimen [6–8] with adverse consequences for both graft and patient survival. Some aspects of HBIG administration can be particularly burdensome for patients. Notably, intramuscular (IM) administration can cause pain and discomfort. Evaluating the impact of such issues is important when assessing quality of life under different HBV prophylaxis regimens.

Until recently, HBIG was available only in intravenous (IV) or IM formulations. The two modalities differ with respect to dosing, timing, schedules and side effects, and can have different impacts on patients’ quality of life and satisfaction with treatment [9–14]. Recently, a new subcutaneous (SC) HBIG formulation became available (Zutectra®, Biotest AG, Dreieich, Germany) [15–17]. The subcutaneous route offers the possibility of self-administration, avoiding the costs incurred by clinic-based administration and the pain of intramuscular injection.

The Immunoglobulin Therapy after Liver Transplantation Questionnaire (ITaLi-Q) is a specific questionnaire developed for assessing the impact of the HBIG therapy on daily activities and patient satisfaction [18]. The questionnaire was tested and validated in a cross-sectional study of patients treated with either IV or IM HBIG, and was found to meet the requirements for internal consistency, reproducibility and discriminatory power required for use in clinical settings [18].

The current study aimed to evaluate changes in quality of life and patient satisfaction when LT recipients were switched from IV or IM HBIG formulations to the SC formulation.

## Methods

### Study design

This was a multicentre, six-month, observational study conducted at nine LT centers in Italy. The protocol was approved by the ethics committees of all participating centers. All patients provided written informed consent.

### Study population

LT patients were eligible for the study if the treating physician, based on clinical judgment, decided to switch from IV or IM HBIG formulations to the SC formulation. Patients were required to be aged 18 years or older, to have undergone LT at least 1 year prior to study entry, to have received the same HBIG formulation (IV or IM) for at least the preceding 6 months, and to have sufficient cognitive capacity to understand the aims of the study and fill out the questionnaire. Patients were ineligible if they had any serious health condition that substantially reduced life expectancy, or any disease or condition that in the opinion of the investigator could interfere with completion of the study.

### Questionnaires

ITaLi-Q includes 37 items and covers eight domains (Table 1) [18]. Responses were based on a 5-point Likert scale. All crude scores were expressed as values between 0 and 100, with higher scores indicating a higher level of the dimension investigated.

**Table 1:** The ITaLi-Q questionnaire

Domain	No. of items	Range	Description
Side effects	4	0–100	A higher score indicates a higher frequency of HBIG-related side effects
Side effects impact	3	0–100	A higher score indicates a higher impact of HBIG-related side effects on daily activities
Negative feelings	6	0–100	A higher score is associated with more negative, therapy-associated feelings (e.g., anxiety and fatigue)
Positive feelings	5	0–100	A higher score is associated with more positive, therapy-associated feelings (e.g., the perceived importance of one’s own health status)
Flexibility	8	0–100	A higher score corresponds to a more disturbing impact of therapy on daily-life flexibility (e.g., planning work or leisure)
Support	3	0–100	The higher the score, the greater is the level of support received to adhere to therapy (e.g., being reminded or being accompanied to outpatient follow-up visits)
Need for support	3	0–100	The higher the score, the greater is the perceived need for support to adhere to therapy
Satisfaction	5	0–100	A higher score indicates a higher degree of satisfaction related to HBIG therapy (e.g., overall satisfaction, satisfaction with the route of administration, and flexibility)

The SF-36, one of the most widely used measures of health-related quality of life, consists of 36 items covering eight dimensions: physical functioning, role limitations caused by physical health problems, bodily pain, general health perception, vitality, social functioning, role limitations caused by emotional health problems, and mental health [19]. Scores on all subscales are linearly transformed to obtain a possible range of 0–100; higher scores indicate a more favorable physical functioning and psychological well-being. The eight domains may be further aggregated into two summary measures: The Physical Component Summary measure and the Mental Component Summary measure. These aggregated scores are transformed to norm-based scores (mean, 50; standard deviation, 10), with higher scores indicating more favorable physical functioning/psychological well-being [20].

### Data collection

Patients completed the ITaLi-Q and SF-36 questionnaires at two different time points: (i) prior to switching from IV or IM HBIG to SC HBIG and (ii) 6 months after switching. The questionnaires were handed to each patient in a sealed envelope for self-administration in an anonymous manner. Pre-specified clinical and sociodemographic data were captured for each patient and correlated with questionnaire results using a univocal numerical code.

### Statistical analysis

Patient characteristics were described with means and standard deviations (SD) or medians and ranges (continuous variables) or with numbers and percentages (categorical variables). Patient characteristics were compared between the subpopulations receiving IV or IM HBIG at baseline using the Mann-Whitney U-test for continuous variables and the chi-square test or the Fisher exact test for categorical variables. Questionnaire scores at 6 months were compared to those at baseline using the Wilcoxon signed ranks test.

No formal a priori sample size estimation was performed. Instead, a sample of consecutive cases seen during a period of 12 months was identified. The actual number of patients enrolled (*N* = 86) provided a 99% power to detect an effect size ≥0.5 for pre-post differences across scores and a power of 79% to detect an effect size ≥0.3. An effect size of 0.5 is generally considered to be the minimum clinically relevant difference [21].

## Results

In total, 86 patients were enrolled, of whom 59 (68.6%) were receiving IM HBIG before switching to SC HBIG and 27 (31.4%) patients were receiving IV HBIG. Demographics and other characteristics were similar between the IM and IV subpopulations at baseline other than a higher proportion of patients working full-time or part-time among those treated with IM HBIG, and a non-significant trend to a higher proportion living alone than in the patients treated with IV HBIG (Table 2).

**Table 2:** Patient characteristics. Data are shown as mean ± SD or counts (%)

Characteristic	Total ***N*** = 86	IM HBIG ***N*** = 59	IV HBIG ***N*** = 27	***P*** value*
Gender				0.13
Male	68 (79.1)	44 (74.6)	24 (88.9)	
Female	18 (20.9)	15 (25.4)	3 (11.1)	
Age	56.6 ± 10.3	55.8 ± 10.8	58.4 ± 9.0	0.41
Occupation				0.04
Employed, full time/part time	25 (29.1)	21 (35.6)	4 (15.4)	
Unemployed	6 (7.0)	4 (6.8)	2 (7.7)	
Retired	41 (47.7)	25 (42.4)	16 (61.5)	
Housewife	11 (12.8)	9 (15.3)	2 (7.7)	
Other	3 (3.5)	0 (0.0)	2 (7.7)	
Years of school education				0.58
≤ 5 years	26 (30.2)	18 (31.0)	8(30.8)	
6–8 years	27 (31.4)	18 (31.0)	9 (34.6)	
9–13 years	27 (31.4)	18 (31.0)	9 (34.6)	
> 13 years	4 (4.7)	4 (4.9)	0 (0.0)	
Not reported	2 (2.3)	1 (1.7)	1 (3.7)	
Marital status				0.20
Single	15 (17.4)	13 (22.0)	2 (7.7)	
Married	61 (70.9)	39 (66.1)	22 (84.6)	
Divorced/widowed	9 (10.6)	7 (11.9)	2 (7.6)	
Not reported	1 (1.2)	0 (0.0%)	1 (3.8)	
Living alone	13 (15.1)	12 (20.7)	1 (3.8)	0.06
Years after LT	8.5 ± 5.9	8.6 ± 5.9	8.3 ± 6.1	0.72
Previous hepatocellular carcinoma	31 (36.0)	21 (36.8)	10 (37.0)	0.98
Diabetes	18 (20.9)	13 (22.0)	5 (18.5)	0.71
Hypertension	31 (36.0)	22 (37.3)	9 (33.3)	0.72
Obesity	11 (12.8)	10 (16.9)	1 (3.7)	0.16

*HBIG* hepatitis B immunoglobulin, *IM* intramuscular, *IV* intravenous, *LT* liver transplantation

*P value for IM versus IV HBIG (Mann-Whitney U-test for continuous variables; chi square or Fisher exact test for categorical variables)

**Fig. 1:**
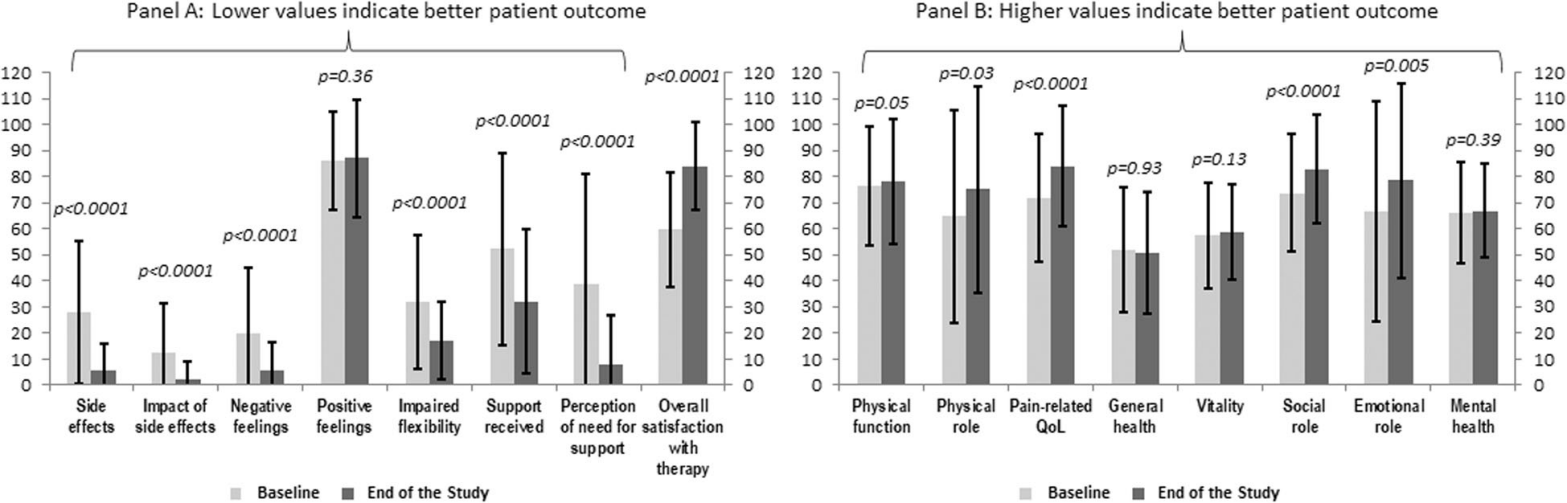
ITaLi-Q (panel **a**) and SF-36 (Panel **b**) scores at baseline and 6 months after the start of SC HBIG (Wilcoxon signed rank test) HBIG, hepatitis B immunoglobulin; MCS, mental component summary score; PCS, physical component summary score; SC, subcutaneous

ITaLi-Q scores at baseline and at 6 months after switching to SC HBIG showed a significant improvement in all the domains other than the item referring to positive feelings (Table 3). All statistically significant changes in scores corresponded to effect sizes over 0.5, the threshold considered to be clinically relevant. The most pronounced improvements were observed for reduction of side effects, need for support in order to adhere to the therapy and overall satisfaction for the HBIG therapy (Fig. 1, panel a).

**Table 3:** Quality of life scores before and 6 months after switch from IV or IM HBIG to SC HBIG

Domain	N	Baseline	End of study	***P*** value*	Effect size
**ITaLi-Q**					
Side effects	83	27.9 ± 27.5	5.5 ± 10.6	**< 0.0001**	0.81
Side effects impact	82	12.6 ± 19.0	2.3 ± 6.7	**< 0.0001**	0.54
Negative feelings	81	19.8 ± 25.5	5.7 ± 10.7	**< 0.0001**	0.55
Positive feelings	81	86.3 ± 18.3	87.0 ± 22.5	0.36	0.08
Flexibility	79	31.8 ± 25.8	17.0 ± 14.9	**< 0.0001**	0.57
Support	75	52.3 ± 36.7	32.0 ± 27.6	**< 0.0001**	0.55
Need for support	80	38.7 ± 42.1	7.7 ± 19.0	**< 0.0001**	0.74
Satisfaction	80	59.6 ± 22.0	84.1 ± 17.1	**< 0.0001**	1.11
**SF-36**					
Physical functioning	85	76.2 ± 22.8	78.2 ± 24.0	**0.05**	0.09
Role physical	81	64.8 ± 41.0	75.0 ± 39.7	**0.03**	0.25
Pain	86	71.7 ± 24.6	83.9 ± 23.1	**<.0001**	0.50
General health	84	51.8 ± 24.0	50.8 ± 23.6	0.93	0.04
Vitality	83	57.4 ± 20.3	58.6 ± 18.2	0.13	0.06
Social functioning	86	73.8 ± 22.7	82.8 ± 20.9	**< 0.0001**	0.40
Role emotional	81	66.7 ± 42.1	78.6 ± 37.4	**0.005**	0.28
Mental health	83	66.0 ± 19.3	67.0 ± 17.9	0.39	0.05
Physical component summary score	75	45.7 ± 8.9	48.1 ± 8.3	**0.002**	0.27
Mental component summary score	75	46.3 ± 10.7	48.2 ± 9.5	**0.007**	0.18

*HBIG* hepatitis B immunoglobulin, *IM* intramuscular, *ITaLi-Q* Immunoglobulin Therapy after Liver transplantation Questionnaire, *IV* intravenous, *SC* subcutaneous

* Wilcoxon signed ranks test

The changes detected by the treatment-specific ITaLi-Q instrument were reflected in broader aspects of quality of life, as assessed by the SF-36. Switching to SC HBIG was associated with statistically significant improvements in physical functioning, role limitations due to physical problems, pain, social functioning, and role limitations due to emotional problems (Table 3). The physical and mental summary scores also increased significantly (Fig. 1, panel b). When the analysis was stratified by baseline HBIG modality, all domains on ITaLi-Q showed a significant improvement 6 months after switching from IM HBIG to SC HBIG, other than the domain for positive feelings (Table 4). General quality of life, as assessed by SF-36, also improved with significant increases in both summary scores (Table 4). In contrast, switch from IV HBIG to SC HBIG was associated with significant improvements for only the ITaLi-Q domains for flexibility and the level of support needs to comply with the IM HBIG regimen, and overall quality of life was not affected (Table 4).

**Table 4:** Quality of life scores before and 6 months after switch from IV or IM HBIG to SC HBIG, stratified by baseline HBIG modality

	IM HBIG			IV HBIG		
Domain	Baseline	End of study	*P* value*	Baseline	End of study	*P* value*
**ITaLi-Q**						
Side effects	38.1 ± 26.1	4.5 ± 9.5	**< 0.0001**	6.9 ± 16.5	7.6 ± 12.4	0.48
Side effects impact	16.8 ± 19.3	2.7 ± 7.5	**< 0.0001**	3.5 ± 14.9	1.6 ± 4.7	0.89
Negative feelings	25.7 ± 27.8	5.3 ± 10.9	**< 0.0001**	6.5 ± 11.5	6.7 ± 10.5	0.81
Positive feelings	84.9 ± 17.3	86.6 ± 21.6	0.28	89.6 ± 20.5	87.9 ± 24.9	0.64
Flexibility	36.9 ± 27.0	17.9 ± 14.4	**< 0.0001**	19.2 ± 17.5	14.9 ± 16.1	**0.05**
Support	55.0 ± 38.5	29.6 ± 24.8	**< 0.0001**	46.4 ± 32.4	37.3 ± 33.0	**0.02**
Need for support	51.9 ± 43.2	7.4 ± 19.0	**< 0.0001**	9.6 ± 19.2	8.3 ± 19.4	0.60
Satisfaction	53.7 ± 21.1	86.5 ± 16.9	**< 0.0001**	72.6 ± 18.3	79.0 ± 16.9	0.25
**SF-36**						
Physical functioning	75.2 ± 21.6	76.1 ± 23.5	0.18	78.6 ± 25.4	82.7 ± 24.8	0.08
Role physical	62.9 ± 40.7	74.6 ± 39.2	**0.02**	69.0 ± 42.3	76.0 ± 41.8	0.72
Pain	68.1 ± 24.3	83.7 ± 23.0	**< 0.0001**	79.7 ± 23.5	84.3 ± 23.8	0.21
General health	45.1 ± 22.2	45 ± 22.7	0.69	65.9 ± 21.7	63.1 ± 21.0	0.31
Vitality	54.1 ± 19.4	57.1 ± 15.8	**0.05**	64.6 ± 20.9	61.9 ± 22.8	0.83
Social functioning	69.7 ± 22.8	81.6 ± 22.4	**0.001**	82.9 ± 20.3	85.6 ± 17.2	0.35
Role emotional	58.8 ± 44.0	75.8 ± 39.8	**0.002**	83.3 ± 33.0	84.6 ± 31.6	0.89
Mental health	63.4 ± 18.7	65.8 ± 16.3	0.22	71.7 ± 19.6	69.5 ± 21.2	0.61
Physical component summary score	44.9 ± 8.0	47.85 ± 7.2	**0.007**	47.4 ± 10.4	49.4 ± 10.1	0.14
Mental component summary score	44.1 ± 10.1	47.8 ± 8.9	**< 0.0001**	50.8 ± 10.5	49.0 ± 10.7	0.22

*HBIG* hepatitis B immunoglobulin, *IM* intramuscular, *ITaLi-Q* Immunoglobulin Therapy after Liver transplantation Questionnaire, *IV* intravenous, *SC* subcutaneous

* Wilcoxon signed ranks test

## Discussion

### Major findings

To our knowledge this is the first study to perform a detailed analysis of quality of life and treatment satisfaction of LT patients following the switch from IM HBIG or IV HBIG to SC HBIG. The two validated instruments used in the study covered both treatment-specific and general health-related quality of life domains, permitting a reliable assessment of the impact of therapy on daily life.

Results from the ITaLi-Q instrument showed that switching from IM or IV HBIG to SC HBIG was associated with a marked reduction in the perceived frequency of HBIG-related side effects, in the impact of such side effects on daily activities, and in negative feelings associated with HBIG use, such as anxiety or fatigue. The SC route of administration also reduced the impact of treatment on the flexibility of daily activities and increased patient autonomy, as documented by the striking decrease in the support required to comply with the scheduled administration of HBIG. These positive effects translated into a marked increase in treatment satisfaction, which in turn could contribute to long-term adherence with the treatment regimen. Switching to subcutaneous HBIG also exerted positive effects on broader aspects of quality of life, encompassing physical functioning, psychological well-being, and social functioning. Quality of life improvement was largely restricted to switch from SC HBIG, mostly likely because pain during intramuscular injections and the need for support for IM administration adversely affects quality of life and everyday activities. Switch from IV HBIG, administered in the hospital setting, somewhat surprisingly resulted in relatively minor improvements in quality of life. Results showed that benefits were seen only for flexibility of daily activities and the need for support to comply with the scheduled administration of HBIG, consistent with the fact that clinic visits were no longer required after introduction of SC HBIG.

From a methodological standpoint, the study shows that the ITaLi-Q instrument, in addition to its very good psychometric properties previously documented [18], is also able to capture longitudinal changes in the impact of the HBIG therapy on daily activities and patient satisfaction.

### Comparison with existing data

Few studies have investigated the impact of different HBIG modalities on quality of life and satisfaction in LT patients. Previously, we reported that patients treated with IM HBIG had significantly higher scores for the ITaLi-Q flexibility and the negative feelings domains than patients given IV HBIG, but lower scores for the side effects domain [18]. A small study in 12 patients conducted at the Vancouver General Hospital investigated the effect of conversion from IM HBIG to SC HBIG. On a pain rating scale of 0 to 10, patients rated their previous IM injections as 5, whereas SC administration was rated as 1.6 [22, 23]. Moreover, all patients reported a preference for SC administration versus IM administration. More recently, a prospective, observational study assessed compliance and tolerability in maintenance LT patients self-administering SCs HBIG at home according to local practice [24]. Patient compliance was graded good or very good by physicians in 91.8% of cases, and treatment was well-tolerated, with no interruption or discontinuation of treatment due to adverse events.

To our knowledge, no data exist on the impact on quality of life of converting from IV HBIG to SC HBIG.

### Implications for clinical practice

Study results could have important implications for clinical practice. Increasing the acceptability of HBIG therapy can help to increase treatment compliance and reduce the risk of post-transplant HBV reinfection. The current findings indicate that the SC route of administration markedly improves the acceptability of treatment, by reducing side effects and their interference with daily life, ameliorating negative feelings, and increasing patient autonomy. Reducing the need for in-hospital administration of HBIG by switching to SC therapy would also be expected to lower health care costs. The striking improvement in treatment satisfaction further suggests that SC HBIG can represent the treatment of choice for most LT patients.

### Study limitations

The study has limitations. First, patients who were proposed to switch from the previous modality of HBIG administration to the subcutaneous modality could be those reporting higher perceived HBIG-related side effects. Second, we have no information on those patients who were proposed the new route of administration, but refused to use it. Therefore, our study population could be not fully representative of patients treated with HBIG. Despite these limitations, our study shows that many patients can derive a substantial benefit from switching to SC therapy.

## Conclusions

In chronic diseases, health-related quality of life is an important measure of the impact of a disease and the effects of medical intervention. An improvement in quality of life is considered to be an essential outcome measure and a determinant of therapeutic benefit. Information on the impact of chronic diseases on patient life is also fundamental to make health services more patient-centered.

With life expectancy after LT now substantially longer than in the past, maximizing the physical and mental health-related quality of the extra years of life is an important priority for LT patients and their relatives. Minimizing the side effects of chronic treatments and their interference with daily life can represent an effective tool to increase acceptance of the disease and adherence to the prescribed treatment regimen. This in turn can help improve long-term prognosis, thus establishing a virtuous circle.

## Data Availability

An anonymous version of the datasets used and analysed during the current study are available from the corresponding author on reasonable request.

## Abbreviations

HBIG: Hepatitis B immunoglobulin
HBV: Hepatitis B virus
IM: Intramuscular
ITaLi-Q: Immunoglobulin Therapy After Liver Transplantation Questionnaire
IV: Intravenous
LT: Liver transplantation
SC: Subcutaneous
SD: Standard deviation
SF-36: Short Form 36 Health Survey
